# Urinary Metabolomic Profile is Minimally Impacted by Common Storage Conditions and Additives

**DOI:** 10.1007/s00192-025-06069-2

**Published:** 2025-02-24

**Authors:** Kelly C. Weldon, Morgan Panitchpakdi, Andrés Mauricio Caraballo-Rodríguez, Alan J. Wolfe, Pieter C. Dorrestein, Linda Brubaker, Lindsey A. Burnett

**Affiliations:** 1 School of Medicine, University of California Davis Health, Sacramento, CA 95817 USA; 2 Skaggs School of Pharmacy and Pharmaceutical Sciences, University of California San Diego, La Jolla, CA 92093 USA; 3 Collaborative Mass Spectrometry Innovation Center, University of California San Diego, La Jolla, CA 92093 USA; 4 Department of Microbiology and Immunology, Loyola University Chicago, Maywood, IL USA; 5 Division of Urogynecology and Reconstructive Pelvic Surgery, Department of Obstetrics, Gynecology, and Reproductive Sciences, UC San Diego, La Jolla, CA USA

**Keywords:** Urinary metabolomics, Urine storage and preservation, Urinary metabolites, Urinary metabolome

## Abstract

**Background:**

Metabolomics reflects the molecular communications within biological systems. Urine is a noninvasive biofluid, rich in metabolites that serve as potential biomarkers for human health and disease. The impact of storage conditions and DNA stabilizers for urine samples in metabolomic studies remain unclear.

**Objective:**

To evaluate the impact of common storage conditions and the presence of a DNA stabilizer, AssayAssure® (Thermo Scientific), on the metabolite content of voided human urine.

**Methods:**

We assessed the urinary metabolite composition under different storage conditions and with the addition of AssayAssure® to determine its effect on metabolomic analysis.

**Results:**

Urinary metabolite composition remained consistent across different storage conditions. However, the addition of AssayAssure® significantly altered the metabolic profile due to adduct formation. Despite these alterations, the identification of parent metabolites was not compromised, and biological differences were still distinguishable.

**Conclusion:**

These findings suggest that urine biobanked under the tested storage conditions is suitable for metabolomic analysis. The addition of AssayAssure® does not hinder the detection of parent metabolites, although it may affect the overall metabolic profile.

**Supplementary Information:**

The online version contains supplementary material available at 10.1007/s00192-025-06069-2.

## Importance

Previous research has demonstrated the significance of the metabolome in other ecological niches, such as the gut and vagina. Many studies have examined the role of the microbiome in urinary pathology; however, limited data exists to suggest whether these samples may also be useful for metabolomic analysis. We analyzed the urinary metabolome in a few common storage conditions and in the presence and absence of the DNA stabilizer, AssayAssure®. As a proprietary product, the exact contents of AssayAssure® are not released to the public, but it is a well-known and commonly used stabilizer for urine samples [1, 2]. Our data indicate biological variability and metabolite composition is maintained among storage conditions and with the addition of AssayAssure®.

## Introduction

The discovery of novel biomarkers is critical to understanding disease states and progression [3]. Metabolomics is the study of metabolic profiles in biological systems and represents the cumulative interaction of genetic, physiologic, and environmental interactions [4, 5]. Unlike many biofluids, urine can be obtained noninvasively, is rich in metabolites, and reflects systemic alterations in human health and disease [6]. An important consequence of these properties is that urine is suitable for the study of health and disease and urinary metabolomics has the potential to discover novel biomarkers associated with disease states.

There are important considerations for collection and storage of urine for metabolic analysis. Metabolites can be readily altered by time and storage temperature and these alterations can contribute to discrepancies in samples collected under differential conditions [7–10]. In ideal circumstances, urine for metabolomic analysis would be snap frozen and analyzed rapidly. Unfortunately, in most clinical settings this is neither feasible nor practical.

In addition, many of the existing urine biobanks were collected and designed for other applications, such as urinary microbiome research, with the addition of preservatives and additives for those types of analysis. To date, it is unknown how the urine metabolome is impacted by commonly used additives for other analyses such as DNA sequencing.

To determine how urine metabolite content is impacted by storage conditions and additives, we obtained voided urine and systematically investigated four commonly encountered storage conditions: refrigeration (2–4 h), simulated shipping, frozen at −80 (3 months) and compared this to quick frozen urine. All conditions were also replicated in the presence of the preservative AssayAssure®, a urine DNA preservative frequently used in the microbiome field. Often, urine biobanks for microbiome research are stored in AssayAssure® for future sequencing, necessitating the need for investigation into whether this preservative is compatible with metabolomics analysis as well. All samples were subjected to untargeted metabolomic analysis. The objective of our study was to compare the impact of storage and AssayAssure® addition on the metabolite content of voided urine.

## Materials and Methods

### Study Participants and Urine Sample Collection

After IRB approval, 10 adult women 18 years or older at the University of California San Diego Health’s Women’s Pelvic Medicine Center were invited to participate in this IRB-approved study (Protocol Number: 801735). Informed research consent was obtained from all participants. Women with known anatomic abnormalities of the urogenital tract, with neurologic or immunologic disease, with a history of bladder malignancy, current systemic infection, or known pregnancy were excluded. All participants contributed a midstream, voided urine specimen, which was collected using the standard “clean catch” protocol. Participants were instructed to wash their hands with soap and water, cleanse the peri-urethral area using a sterilizing wipe, discard the initial urine stream, and then collect midstream urine into a sterile Becton Dickinson (BD) Vacutainer cup.

### Urine Sample Preparation

Aliquots (200 μL volume) of midstream voided urines were immediately pipetted into microcentrifuge tubes in triplicate and subjected to the following storage conditions: (1) snap frozen in ethanol bath on dry ice, (2) 4ºC refrigeration for 2–4 h prior to extraction, (3) transport on wet ice and left in insulated box for 24 h prior to extraction to simulate postal shipping methods prior to extraction, and (4) transport on dry ice to the lab followed by storage in −80 for 3 months prior to extraction. AssayAssure® (Thermo Scientific, SKU 14401) was added to half of the samples prior to simulated storage.

### Metabolomics Sample Preparation

At designated timepoints sample aliquots were extracted by adding 100% MeOH with 1 μM of sulfamethazine as a synthetic internal standard, to achieve a final concentration of 80% MeOH. The samples were vortexed then incubated at −20°C for 20 min to aid in protein precipitation. The samples were then centrifuged for 15 min at 14,000 rpm and 80% of the supernatant 800 μL was transferred to a prelabeled 96-Well DeepWell plate. The plates were concentrated using a CentriVap Benchtop Vacuum Concentrator until dry. Plates were covered and stored at −80°C until data acquisition.

For data acquisition, the plates were resuspended in 250 μL of a 50% MeOH to water solution containing 1 μM of sulfadimethoxine as an internal standard. Untargeted metabolomics data were collected using an ultra-high-performance liquid chromatography system (Vanquish, Thermo Fisher Scientific, Waltham, MA, USA) with a Kinetex 1.7 µm 100 A pore size C18 reversed phase UHPLC column 50 × 2.1 mm (Phenomenex, Torrance, CA, USA) coupled to an orbitrap mass spectrometer (Q-Exactive Hybrid Quadrupole-Orbitrap, Thermo Fisher Scientific, USA). The mobile phase solvent A was water with 0.1% formic acid, and the mobile phase solvent B is acetonitrile with 0.1% formic acid (LC–MS grade solvents, Fisher Chemical, USA). The flow rate was set to 0.500 mL/min and a 10 min per sample run time was used with a linear gradient as follows: 0 to 1 min, constant at 5% B; 1 to 7 min, linear increase to 99% B; 7 to 8 min, constant at 99% B; 8 to 8.5 min, linear decrease to 5% B; 8.5 to 10 min, constant at 5% B. Positive mode electrospray ionization was used.

### Metabolomics Data Processing and Analysis

Data in the .raw format was converted to .mzXML files using the ProteoWizard tool MSConvert. All data (.raw and.mzXML) was uploaded to MassIVE, saved under the ID MSV000091929 (ftp://massive.ucsd.edu/MSV000091929/). MS1 feature finding was conducted using the GNPS LC MZmine2 workflow, set to the preexisting batch mode: “Thermo QExactive – Kelly SEED – Mzmine-2.53)” and MZmine2 version 2.53. Three MZmine jobs were conducted for this study with these parameters: a first job including all samples, blanks and QC samples, a second job including just the samples contained in the AssayAssure® solution, and a third job of just the samples not stored in AssayAssure®. Three jobs were conducted as the presence of the AssayAssure® altered the parameters of MZmine; therefore, to understand the metabolite presence in samples without AssayAssure®, the separate jobs were necessary. All three MZmine feature tables, along with sample metadata, were then input into the global natural products social (GNPS) feature-based molecular networking (FBMN) tool [11] for molecular networking and library ID generation, using all publicly available libraries and suspect libraries [12]. The .qza files produced from GNPS and sample metadata were used as inputs for QIIME 2 [13] to perform the beta diversity analysis of the data, principal component analysis (PCoA) and biplots, and random forest analyses (https://docs.qiime2.org/2023.9/). Finally, the .graphml files were downloaded from the GNPS FBMN jobs and input into the free Cytoscape [14] software to visual the molecular networks.

### Statistical Analysis

For global metabolome comparisons, PERMANOVA calculations were performed on Bray Curtis distance matrices using QIIME 2 with *p* < 0.05 as significant. For comparison of relative abundance of individual metabolites, Kruskal–Wallis nonparametric analysis followed by Dunn’s multiple comparison tests were performed with *p* < 0.05 as significant.

## Data Citation and Availability

UCSD MassIVE ID: MSV000091929.

ftp download: ftp://massive.ucsd.edu/v07/MSV000091929/

Full dataset GNPS Feature-Based Molecular Networking: https://gnps.ucsd.edu/ProteoSAFe/status.jsp?task=d434e978d972449b89eeeefd7c8d1816

AssayAssure® Dataset GNPS Feature-Based Molecular Networking: https://gnps.ucsd.edu/ProteoSAFe/status.jsp?task=4366f3b00e7c44219affec6f1dd0f91e

Non-AssayAssure® Dataset GNPS Feature-Based Molecular Networking: https://gnps.ucsd.edu/ProteoSAFe/status.jsp?task=b810c03d02fa404d8bdace84be8ccf99

Shannon, P. et al. 2003. Visualization Software Cytoscape.10.1101/gr.1239303. Retrieved January, 2021. {*Code and/or software.*

Bolyen, E. et al. 2019 *Nat. Biotech*. QIIME 2. 10.1038/s41587-019-0190-3. . Retrieved January 2021. {*Code and/or software.*}

## Results

### Urine is Rich in Metabolites

Untargeted metabolomics data was acquired and analyzed for 240 urine samples (4 storage conditions, ± AssayAssure®, 10 participants, in triplicate). Overall, the entire dataset generated 6259 MS2 features using the GNPS MZmine2 workflow and 274 library matches through GNPS FBMN; 3303 metabolites were networked to at least one other metabolite; 747 metabolite networks were identified which ranged from 2 to 94 metabolites in size. Of the 747 molecular families identified, all but two contained metabolites found in both the presence and absence of AssayAssure® (AA).

### Individual Participants Drive Data Clustering While Storage Conditions have Minimal Effect

**Fig. 1:**
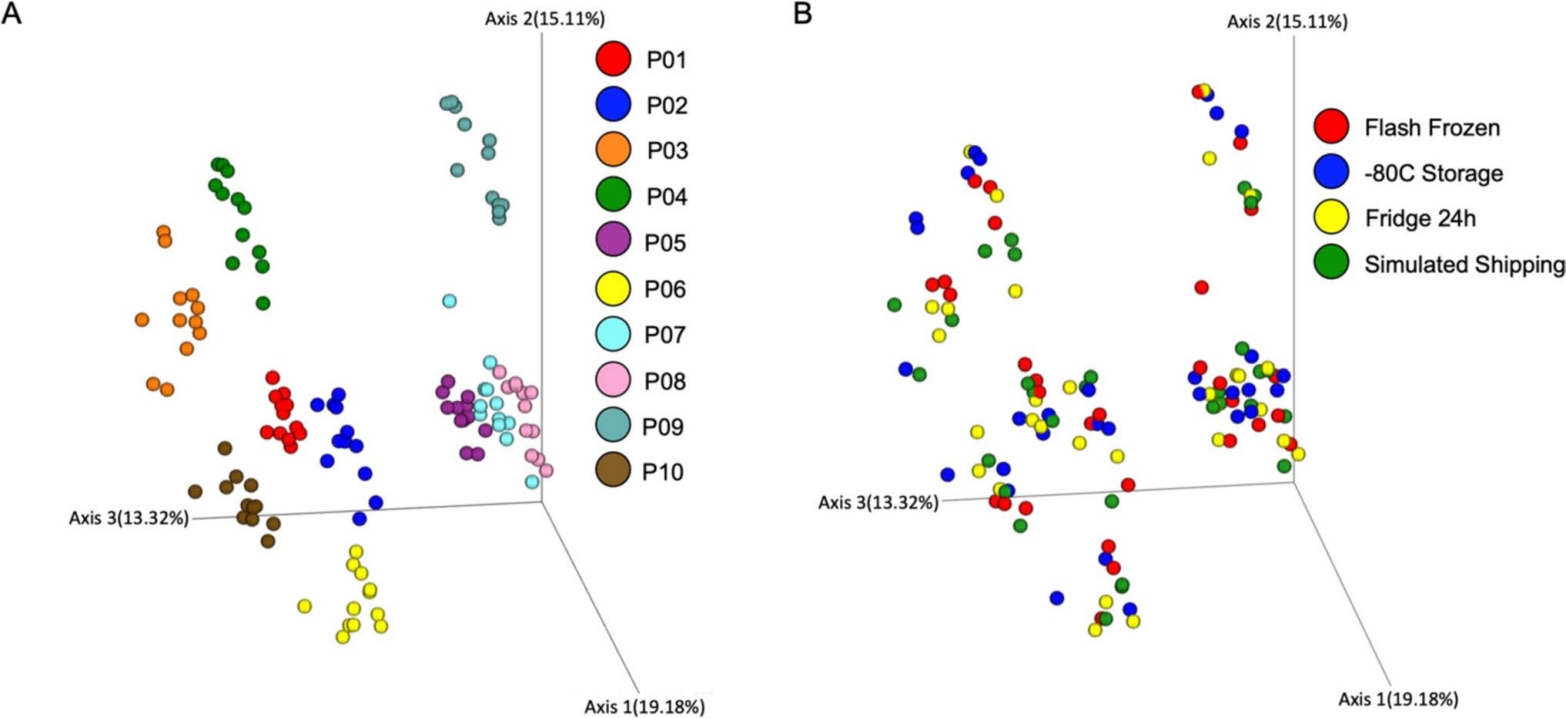
Urinary Metabolomic Profile by Participant and Storage Condition. **A** Principal component analysis (PCoA) calculated using a Bray Curtis distance metric of metabolomics samples without AssayAssure® (non-AA), colored by participant ID showing significant differences in metabolite composition between participants (PERMANOVA *p* = 0.001, pseudo-F = 64.013). **B** Bray Curtis PCoA of metabolomics samples non-AA, now colored by storage method (flash frozen (red), freezer storage (blue), fridge 24 h (yellow), simulated shipping (green)), showing no significant difference between storage conditions (PERMANOVA *p* = 0.999, pseudo-F = 0.359828)

Feature finding in the non-AssayAssure® (non-AA) dataset resulted in fewer MS2 features (4205 metabolites) and annotations (187 library matches) than the combined data set. Of these, 2075 metabolites were networked to at least one other metabolite, resulting in 434 molecular families ranging from 2 to 99 metabolites in size. The beta diversity of the non-AA samples revealed a tight clustering by participant ID on the Bray Curtis PCoA (Fig. 1A). PERMANOVA analysis on the separation between participants showed a significant difference in the composition of the metabolomics data between participants (*p* = 0.001, pseudo-F = 64.013).

When analyzed by storage condition, no separation or trends were seen due to storage condition on the PCoA plot (Fig. 1B). PERMANOVA analysis also indicated that there was no significant clustering by storage condition (*p* value = 0.999, pseudo-F = 0.359828). Random forest analysis using the QIIME 2 sample-classifier function performed slightly over baseline (accuracy ratio = 1.556), producing an ROC curve result of AUC = 0.65. This performance was mainly due to the fact that the algorithm was able to predict the classification of the 3-month freezer samples with 100% accuracy. This high accuracy may indicate changes in abundance differences of metabolites when frozen for extended periods of time; however, changes were not large enough to see a significant difference between samples’ overall beta diversity, as seen with the nonsignificant PERMANOVA score.

As urine metabolite composition varied significantly among participants, we next examined separate participant tables to analyze the difference in storage conditions for each participant. Individual PCoAs of non-AA samples did not show distinct storage method separation (Example PCoA in Supplemental Fig. 1A) and all participant level PERMANOVA analyses of non-AA samples resulted in insignificant variation between storage methods for every participant (Supplemental Fig. 1B). Overall, for the samples not stored in AA, there were no significant differences in metabolomic profiles between the four tested storage conditions (Flash frozen, 3 months in freezer, 24 h in fridge or shipping simulation).

### Sample Metabolomic Trends are Similar With and Without AssayAssure®

Next, samples stored in AA were analyzed. The GNPS MZmine workflow found 5303 MS2 features and GNPS FBMN identified 238 library matches within these MS2 features; 2640 metabolites were networked to at least one other metabolite, creating 564 networks ranging in size from 2 to 88 metabolites. Overall, the AA samples resulted in 1098 more MS2 features found by the MZmine software and 51 more annotations than the same samples stored without AA. These results may indicate the presence of AA contaminants in the data, as well as additional adducts and mass shifts.

However, the overall metabolomic profile of the data remained similar to what was seen with the non-AA samples. Beta diversity analysis of the data indicated a tight clustering by participant and no obvious trends from storage conditions, as visualized by PCoA (Supplemental Fig. 2A and 2B). PERMANOVA analysis by participant ID resulted in a significant differentiation between participants (*p* = 0.001, pseudo-F = 22.4337). PERMANOVA test of the storage conditions indicated no significant difference between the stored conditions across all participants (*p* = 0.999, pseudo-F = 0.359828). Additionally, the random forest analysis showed an overall predictive accuracy of 0.694444, which is 2.77778 times the baseline accuracy. The algorithm was able to classify the test samples from the flash frozen group with 100% accuracy, and samples from the freezer 3-month group with 88% accuracy. Overall, this indicates that for AA samples, the type of storage may slightly alter the preservation of certain metabolites. However, similar to the non-AA samples, the machine learning predictive ability does not result from an overall significant beta diversity profile shift of the data, as was demonstrated by the PERMANOVA test.

**Fig. 2:**
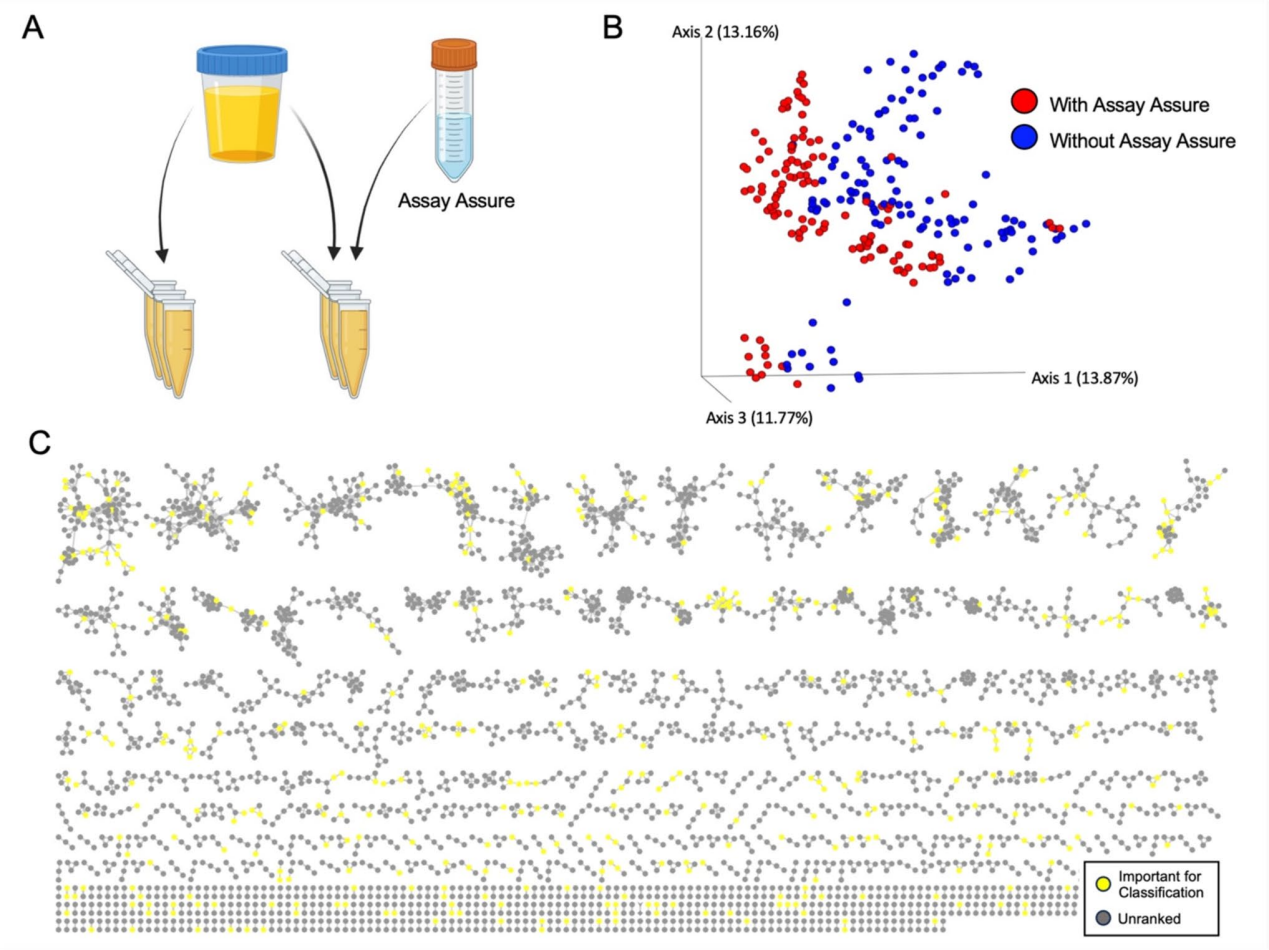
Urinary Metabolomic Profile in Presence/Absence of AssayAssure®. **A** Samples were either stored with or without the addition of DNA preservative, AssayAssure®. **B** Principal component analysis (PCoA) of metabolomics Bray Curtis of samples with (red) and without (blue) AssayAssure® (PERMANOVA *p* = 0.001, pseudo-F = 18.8938). **C** Urinary metabolite GNPS network. Each circle represents a cluster of matches MS2 spectra (features), connected by edges where the width of the line indicates a higher cosine score for spectral alignment (minimum cosine = 0.70). Random forest importance score was used for coloration of clusters, with yellow indicating an importance score of 0.001 or higher

Next, we examined separate participant data to ascertain whether AA differentially impacted metabolite composition by storage condition using the Bray Curtis distance metric. Unlike the non-AA samples, AA samples from half of participants did show significant separation between sample storage conditions as shown in the example PCoA plot and in the table (Supplemental Fig. 1C and 1D). As Bray Curtis was used for the original analysis, we then analyzed the AA samples using the Jaccard metric to assess whether this significant difference was due to changes in metabolite abundance or changes in metabolite presence or absence: only 2 participants showed significant separation in the Jaccard (presence/absence) beta diversity of their samples by storage conditions (*p* = 0.028 and 0.038) while pseudo-F values remained comparable to participants with nonsignificant metabolome separation (pseudo-F = 1.22806 and 1.15052). Overall, this indicates subtle alteration in metabolite abundances rather than complete metabolite depletion based on storage conditions in the presence of AA. The biologic variation between participants superseded the impact of AA and storage conditions similar to non-AA samples.

### AssayAssure® Shifts Entire Metabolome Profile in a Predictable Way

We next examined how AA influences metabolite composition in all samples. The metabolomic composition between AA samples and non-AA samples was visualized by a PCoA plot using a Bray Curtis distance metric (Fig. 2B). Samples showed significant separation AA and non-AA groups (*p* value = 0.001, pseudo-F = 18.8938 by PERMANOVA). Of the 6259 MS2 spectra in the entire dataset, six were unique to non-AA samples and 65 were unique to AA samples. However, of the 747 molecular families identified in the entire dataset, only two doublet networks were unique to AA samples. The remaining unique metabolites (to AA samples) were networked to metabolites present in non-AA samples. These data indicate that, although unique metabolites exist in the presence of AA, these spectra represent modifications to metabolites already present in non-AA samples.

To determine the impact of AA on metabolite abundance, a random forest analysis was performed. The model was able to accurately predict between non-AA and AA samples (accuracy ratio = 1.91667). To visualize how AA changed the metabolite abundance, features that were identified by the analysis important for sample classification were annotated in the molecular network (Fig. 2C). The variable presence of significant features across metabolite networks indicates that metabolite networks were differentially impacted by the presence of AA.

**Fig. 3:**
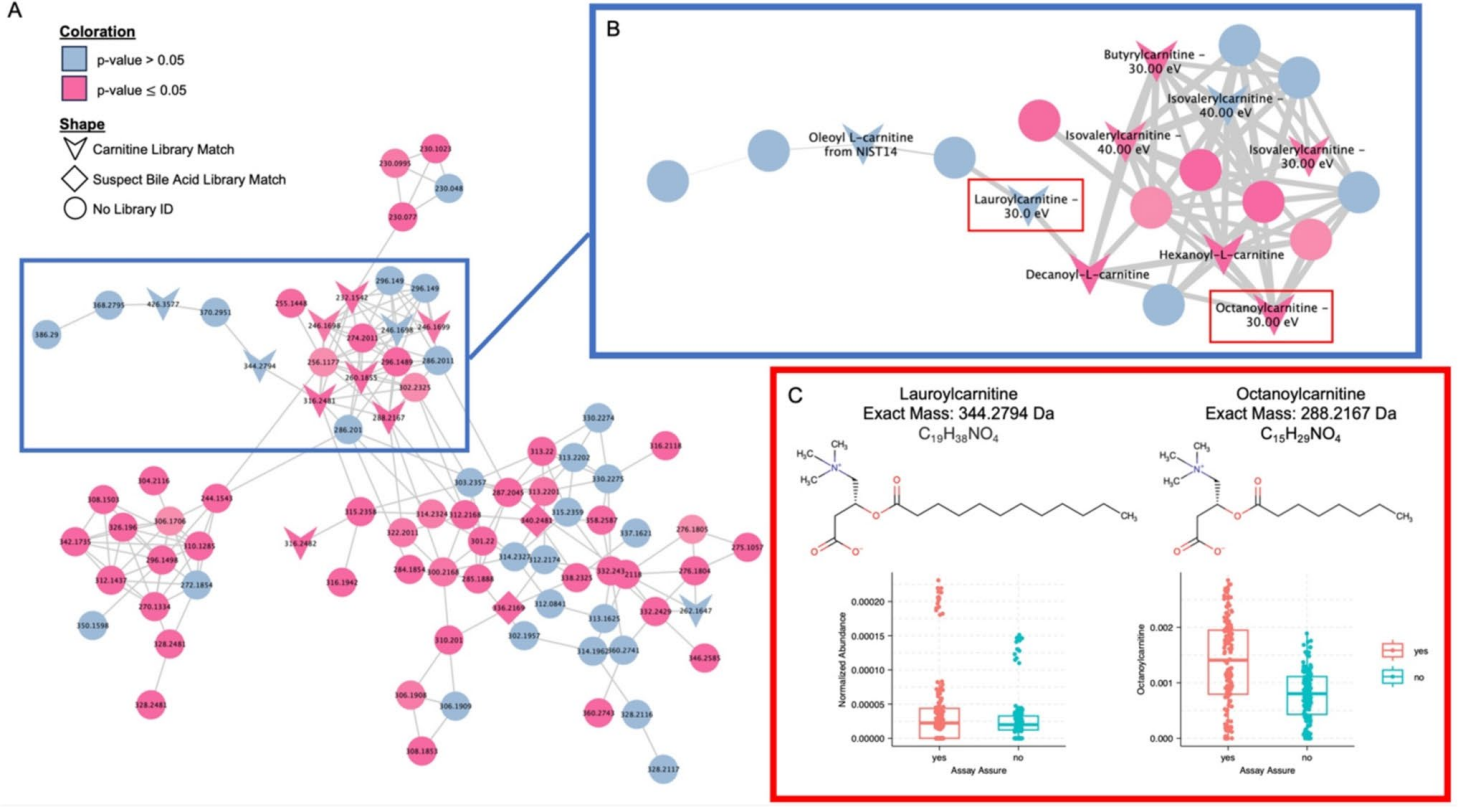
Carnitines are Differentially Preserved in AssayAssure®. **A** The full carnitine molecular family network produced by GNPS FBMN. Each node represents a cluster of aligned spectra, identified as one feature. Nodes are labeled by parent mass (Da), shaped by library annotation status, and colored by a Dunn’s test conducted on the normalized abundance levels between AssayAssure® samples and those without it. Edges between nodes indicate a spectral connection, with a cosine score > 0.70. **B** A zoomed in portion of the larger molecular family (indicated by the blue boxes) where the nodes are now labeled by GNPS Library ID match. Shapes are still indicative of spectral match category; coloration is still by Dunn’s test. The widths of the edges are now indicative of a larger cosine score, with the larger edges being a cosine closer to 1. **C** Two example metabolites from the network, indicated by the red boxes. Structures are presented and the normalized abundance levels are plotted. As with most of the carnitine metabolites, these example metabolites are found in higher abundance in the AssayAssure® samples

To explore the phenomena, we examined one of the largest molecular families containing a majority of the carnitine library matches, a common and biologically relevant urine metabolite [15, 16]. A Dunn’s test was performed across the molecular family and the *p* values comparing AA to non-AA abundance were visualized (Fig. 3A). The carnitine network indicated that multiple metabolites within the same network demonstrated significant abundance differences when stored in AA or without (Fig. 3B). Specific to the carnitines, AA was able to more highly preserve certain metabolites over non-AA (Fig. 3C). Supplemental Table 1 contains a list of every multiple test corrected *p* value along with which group (AA vs non-AA) contained the highest median abundance of the feature. Overall, in this family, 55 of the 85 features were significantly different between the AA versus non-AA samples. Forty-nine of these 55 features were of higher abundance when stored in AA. In addition to the carnitine library matches within this molecular family, there were also a few potential bile acids, which were also significantly more abundant in the AA samples. Although the median abundance levels for many features were higher in the AA samples, these features were still present in the non-AA samples. The addition of AA to the samples slightly altered the preferential preservation of some metabolites but did not completely add or completely lose any features in this molecular family.

To determine whether the addition of AA reduced interparticipant metabolome separation, the entire dataset was analyzed by participant. The PERMANOVA test still found significant variation between individuals (*p* = 0.001, pseudo-F = 40.6167), despite the separation seen between AA and non-AA. All participants can be distinguished from each other using their urine metabolome whether their samples were stored in the presence or absence of AA. Additionally, storage condition was analyzed at this scale, and conditions were not significantly distinct from each other using the PERMANOVA analysis (*p* = 0.755, pseudo-F = 0.840106). Overall, the entire dataset is overwhelmingly separated by participant, and then within each participant cluster, there is a separation by whether the samples were stored in the presence of AA. Other storage conditions had no significant effect on separation when analyzed within the entire dataset.

## Discussion

Broad untargeted metabolomics projects are subject to batch effects due to the collection methods and sensitivity of the data type [17]. Consequently, it is unclear whether metabolomic analysis of stored and preserved urine would yield biologically relevant data. Protocols and best practices have been developed for metabolimcs urine preparation, but these mainly focus on when urine is collected and how storage will preserve very specific known metabolites and biomarkers. Protocols and best practices for preparing urine samples for metabolomics primarily focus on optimizing collection timing and preserving specific known metabolites and biomarkers [18]. While previous studies have examined how storage conditions affect certain urinary metabolites, no comprehensive analysis using untargeted metabolomics has been conducted [7, 8]. This study leverages extensive untargeted metabolomics libraries and advanced metabolite networking techniques to investigate not only changes in the abundance of urinary metabolites but also alterations in adducts and ionization patterns. Here, we assessed whether common storage conditions in the presence or absence of the DNA preservative AssayAssure® (AA) impacted urine metabolomic profiles. There are two principal findings of this work: (1) the storage conditions tested do not significantly impact urine metabolite composition and (2) the presence of AA is not associated with metabolite loss despite significant changes in abundance of some urinary metabolites. In addition, unique metabolites were added by the AA network with metabolites that are seen in urine samples without AA.

This analysis indicated that the effects of the four common storage conditions (refrigeration (2–4 h), simulated shipping on wet ice, frozen at −80°C (3 months), and quick-frozen urine) were minimal to the overall metabolomic profile. Metabolomic profile diversity in both samples with and without AA did not vary by these four storage conditions. The machine learning analysis was able to learn and predict samples into their storage conditions above a baseline random approach, although this separation was not reflected in the overall beta diversity. This finding is consistent with the conclusion that storage conditions did not change the overall urinary metabolite profile of the samples, although the storage conditions may have resulted in the minor differential preservation of a few compounds in which the machine learning was able to identify and predict from. Analysis separated by participant showed no differences between storage conditions, indicating biologically relevant interparticipant variability outweighs the impact of storage conditions or the presence or absence of AA.

The separation between the metabolomics data for sample stored with vs. without AA is significant and not surprising, given the certainty that the preservative solution adds unique features. Interestingly though, this separation is not just due to the presence of AA contamination features but an overall shift in a large amount of feature abundances across the dataset, as seen with the machine learning visualization of the network. AA is used to protect RNA and DNA from degradation within samples by suppressing enzymatic activity, which resulted in increased abundance of some metabolites as well. We illustrated metabolite compositional changes within the carnitine molecular family (Fig. 3 and Supplemental Table 1). Notably, although important metabolites were not lost when using AA, they were present in different abundances and adducts within networks.

Additionally, within the AA samples there was more separation between the four storage conditions than when analyzing the non-AA samples. Five of the ten participant individual datasets indicated a significant separation between the four sample storage conditions. Caution is needed in interpretation, as this small sample sizes has the potential for skewed results. These data suggest preservation in the presence of AA may be dependent on storage conditions. Despite this variability, the interparticipant variability continued to supersede the impact of storage conditions or AA presence. Overall, these data suggest that ideal sample storage and preservative conditions should be matched between participants. It also suggests that the tested conditions do not preclude metabolomic analysis. Finally, caution is recommended when interpreting subtle changes in metabolic abundance longitudinally or with variable storage for a given participant.

In all storage conditions analyzed in the presence or absence of AA, biological metabolites were identified, and biological differences were still significant—indicating that samples from all conditions can be used for metabolomic analysis producing meaningful results. AA is not a background signal; it results in differential preservation of metabolites as well as different adduct formation. Parent molecules are still present in all samples, indicating that it is plausible to use urine samples preserved with AA for metabolomics analysis. This finding suggests biobank samples may be informative for both sequencing and metabolomics (multi-omics) projects.

Strengths of this work are that frequently employed storage and preservative conditions were studied making this analysis applicable to biobanked urine specimens stored under similar conditions. Additionally, this analysis included global profiling of metabolites rather than a targeted approach and is applicable for a wide range of clinically applicable metabolites.

Limitations of this data include the small sample size with a limited participant population that lacks phenotypic or demographic characterization of participants. Furthermore, only a single collection method: midstream, voided urine, was used, which is presumed to represent the urogenital and not urinary only metabolome. Additional studies are needed to determine the relationship between the voided urine metabolome and samples collected using other methods (i.e., catheterized, suprapubic aspirate, etc.). Metabolites in lower abundance in participant populations or exclusively found in specific participant populations may not be represented in the metabolomic profiles used in this work. Consequently, these metabolites may have a divergent response to the conditions and preservative used in this study. In addition, we did not include a prolonged frozen timepoint which may impact metabolite preservation.

## Conclusion

This study evaluated the effects of common urine storage conditions and the DNA preservative AssayAssure® (AA) on metabolomic profiles, demonstrating that storage methods—refrigeration, simulated shipping on wet ice, freezing at −80°C, and quick freezing—did not significantly alter overall metabolite composition. While AA introduced unique adducts and altered the abundance of certain metabolites, parent molecules were preserved, making it feasible to use AA-preserved samples for metabolomic analyses. The minimal impact of storage conditions on metabolite profiles, compared to the significant interparticipant variability, supports the robustness of metabolomic data under these conditions.

To ensure reliability in metabolomics studies, consistent storage protocols across participants are recommended, with freezing at −80°C or quick freezing as optimal methods. While AA enhances metabolite preservation and enables multi-omics analyses, its effects on metabolite abundance and adduct formation warrant careful interpretation. Currently, analysis of samples stored in AA versus samples without should be done separately. Expanding future research to include diverse sample collection methods and prolonged storage durations will further validate these findings. Overall, biobanked urine samples stored under the tested conditions are suitable for metabolomic analyses, offering meaningful insights into biological variability across participants.

## Supplementary Information

Below is the link to the electronic supplementary material.

Supplementary file1 (DOCX 815 KB)
