# Supplementary material for: Urinary Metabolomic Profile is Minimally Impacted by Common Storage Conditions and Additives
Source: Int Urogynecol J. 2025 Feb 24;36(4):839–47. doi: 10.1007/s00192-025-06069-2 (PMC12064589; doi:10.1007/s00192-025-06069-2)
Supplement: Supplementary file 1 — Supplementary file1 (DOCX 815 KB) [file 192_2025_6069_MOESM1_ESM.docx]

**Supplemental Figures/Tables**


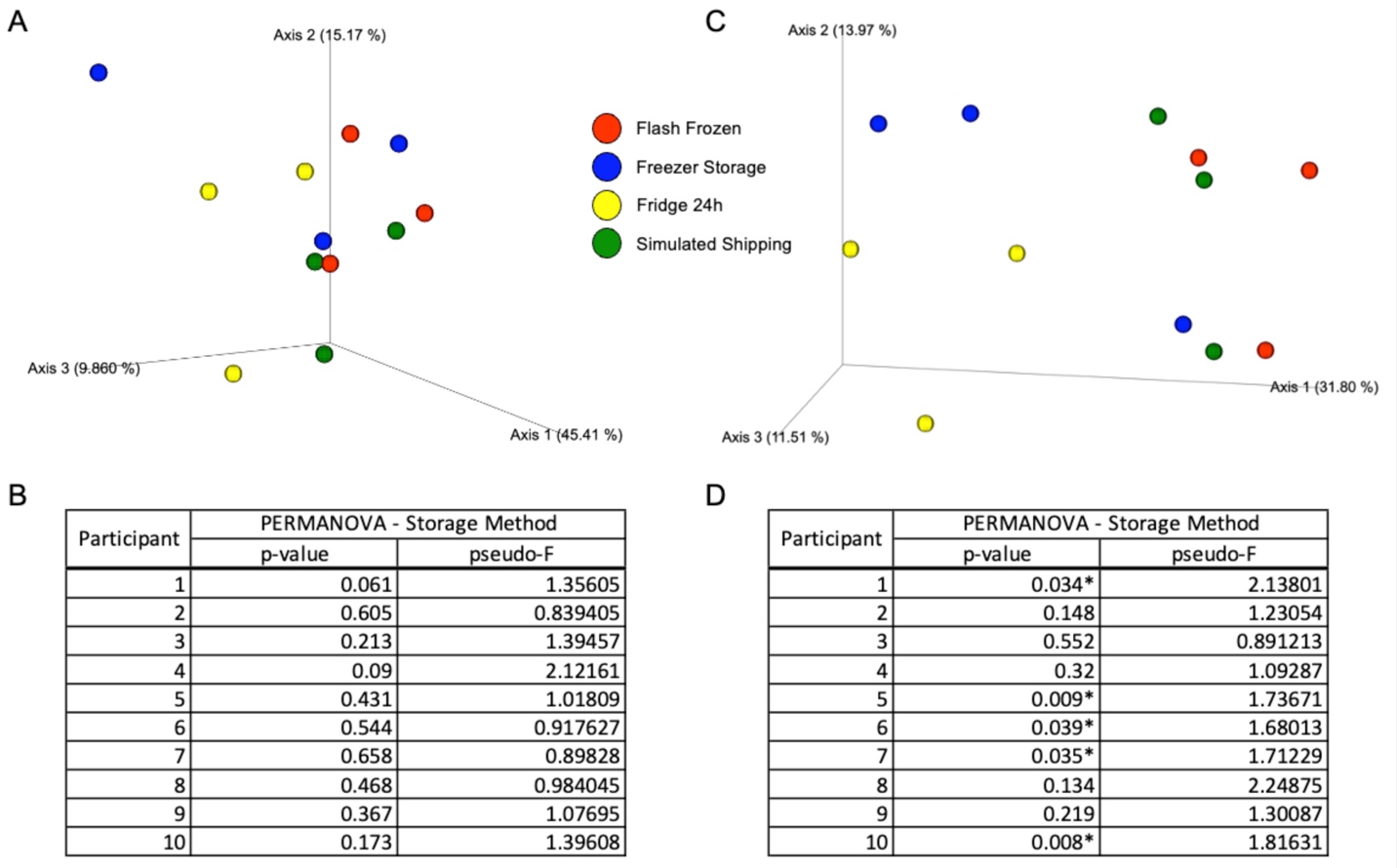


**Supplemental Figure 1**. Example of individual participant storage condition analysis, using Participant 10 data. A. Bray Curtis PCoA plot of Participant 10’s samples stored without AssayAssure®, colored by storage condition. B. Table of the PERMANOVA scores for the samples without AssayAssure® for each individual participant, calculated from the Bray Curtis distance metric. No significant separation between storage condition was seen for any participant. C. Bray Curtis PCoA plot of Participant 10’s samples stored in AssayAssure®, colored by storage condition. B. Table of the PERMANOVA scores for the samples in AssayAssure® for each individual participant, calculated from the Bray Curtis distance metric. Significant variance between storage conditions can be seen 5/10 of the participants for the AssayAssure® stored samples.


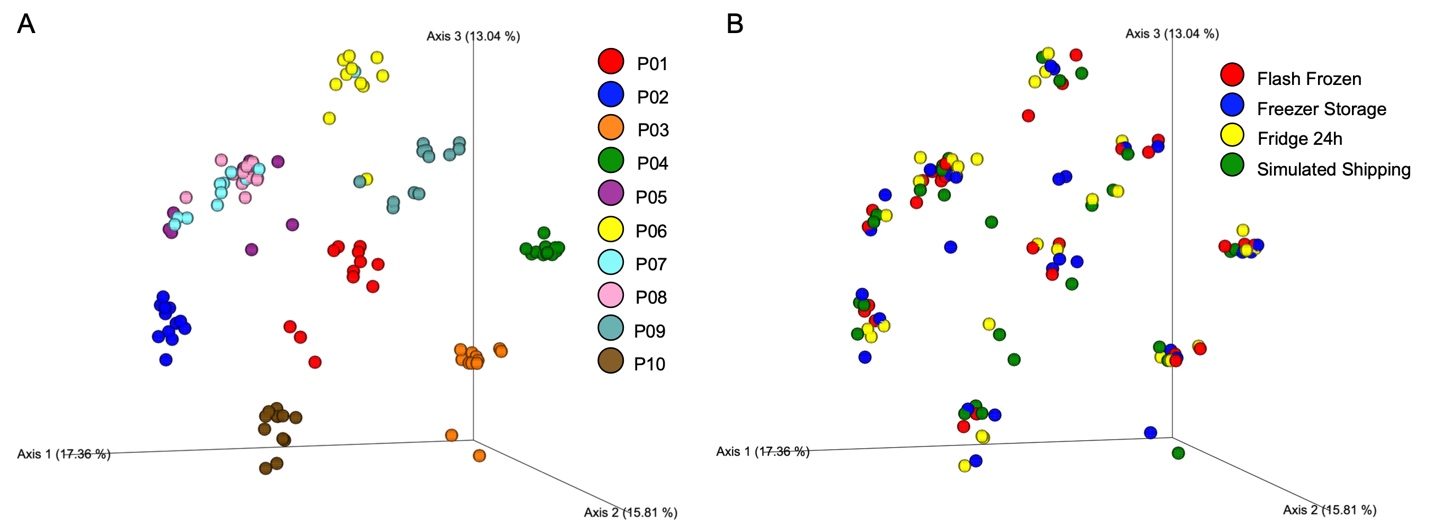


**Supplemental Figure 2**. AssayAssure® stored Urinary Metabolomic Profile mimics samples without AssayAssure®. A. Principal component analysis (PCoA) calculated using a Bray Curtis distance metric of metabolomics samples stored in AssayAssure®, colored by participant ID showing significant differences in metabolite composition between participants (p = 0.001, pseudo-f = 22.4337). B. Bray Curtis PCoA of metabolomics samples stored in AssayAssure®, now colored by storage method (flash frozen (red), freezer storage (blue), fridge 24h (yellow), simulated shipping (green)), showing no significant difference between storage conditions (p = 0.999, pseudo-f = 0.359828).


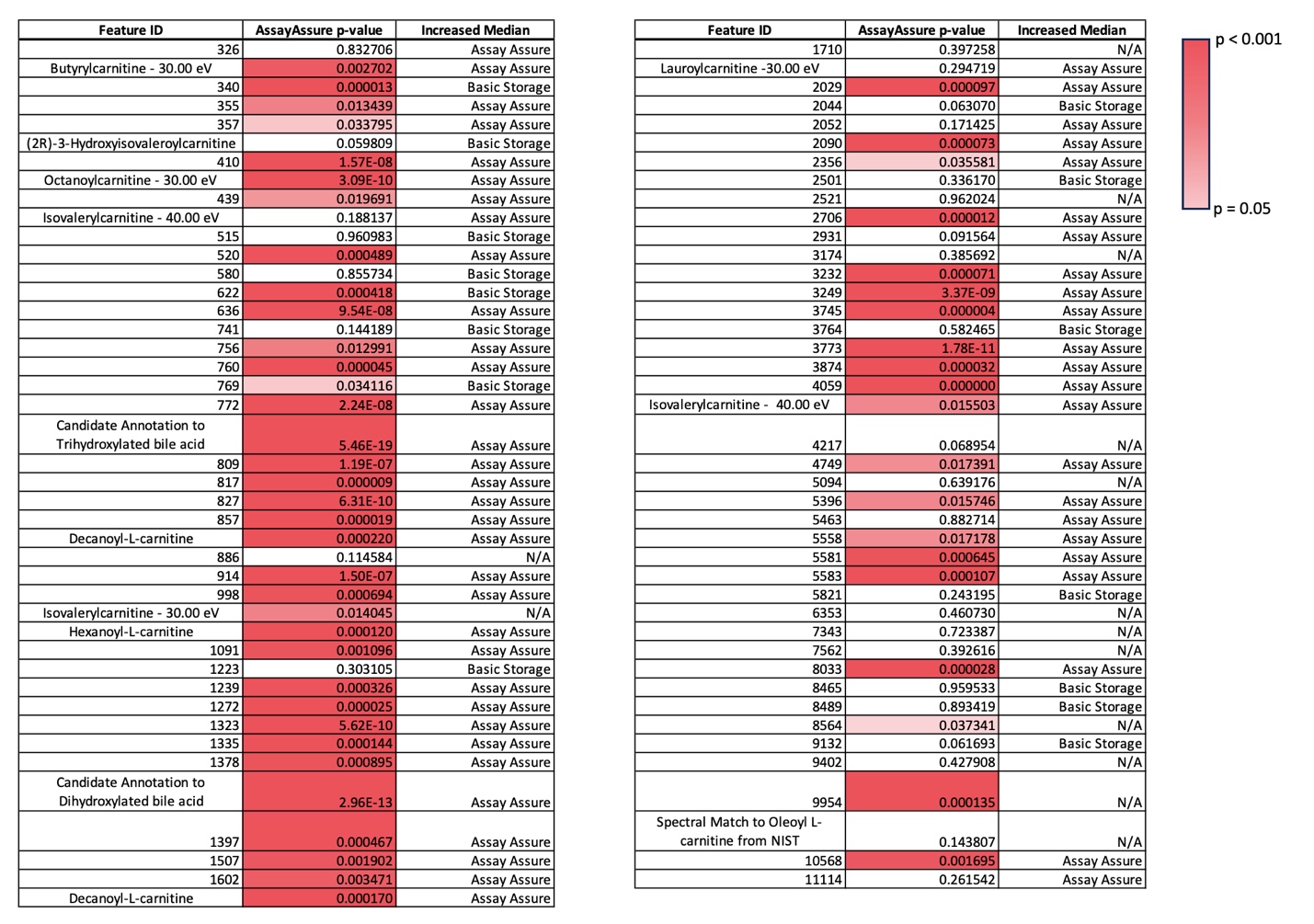


**Supplemental Table 1**. Significant differences in abundance levels seen within the carnitine molecular family. This table contains every feature in the network visualized in Figure 3, labeled by feature ID or library ID if present. The p-values were calculated using a Dunn’s test and highlighted in red if significant (p < 0.05). The final column indicated in which sample type the median of the data is higher, a representation of which sample type the feature is more abundant in.
